# Association of tumor immune infiltration and prognosis with homologous recombination repair genes mutations in early triple-negative breast cancer

**DOI:** 10.3389/fimmu.2024.1407837

**Published:** 2024-07-04

**Authors:** Zheng Wang, Anqi Li, Yujie Lu, Mengyuan Han, Miao Ruan, Chaofu Wang, Xiaotian Zhang, Changbin Zhu, Kunwei Shen, Lei Dong, Xiaosong Chen

**Affiliations:** ^1^ Department of General Surgery, Comprehensive Breast Health Center, Ruijin Hospital, Shanghai Jiao Tong University School of Medicine, Shanghai, China; ^2^ Department of Pathology, Ruijin Hospital, Shanghai Jiao Tong University School of Medicine, Shanghai, China; ^3^ Department of Translational Oncology, Amoy Diagnostics Co., Ltd., Xiamen, China

**Keywords:** triple negative breast cancer, homologous recombination repair genes, mutation spectrum, clinicopathological factors, immune infiltration, prognosis

## Abstract

The aim of this study was to evaluate the mutation spectrum of homologous recombination repair (HRR) genes and its association with tumor immune infiltration and prognosis in triple-negative breast cancer (TNBC). TNBC patients (434 patients from Ruijin cohort) were evaluated with targeted next-generating sequencing for mutations in HRR genes. The frequencies of mutations were compared with public reference cohorts (320 TNBC patients from METABRIC, 105 from TCGA, and 225 from MSKCC 2018). Associations between mutation status and tumor immune infiltration and prognosis were analyzed. HRR genes mutations were seen in 21.89% patients, with *BRCA1/2* mutations significantly enriched in tumors with breast/ovarian cancer family history (*P* = 0.025) and high Ki-67 levels (*P* = 0.018). HRR genes mutations were not related with recurrence-free survival (RFS) (adjusted *P* = 0.070) and overall survival (OS) (adjusted *P* = 0.318) for TNBC patients, regardless of carboplatin treatment (*P* > 0.05). Moreover, tumor immune infiltration and PD-L1 expression was positively associated with HRR or *BRCA1/2* mutation (all *P* < 0.001). Patients with both HRR mutation and high CD8^+^ T cell counts had the best RFS and OS, whereas patients with no HRR mutation and low CD8^+^ T cell counts had the worst outcomes (RFS *P* < 0.001, OS *P* = 0.019). High frequency of HRR gene mutations was found in early TNBC, with no prognostic significance. Immune infiltration and PD-L1 expression was positively associated with HRR mutation, and both HRR mutation and high CD8^+^ T cell infiltration levels were associated with superior disease outcome.

## Introduction

The homologous recombination repair (HRR) pathway is responsible for cell differentiation, DNA mismatch repair, transcriptional regulation, and cell apoptosis in order to maintain genomic stability and suppress oncogenesis (1). *BRCA1/2* are key genes to the HRR process following DNA double strand breaks (DSBs) (2), and other genes involved in the HRR pathway include *ATM*, *BARD1*, *PALB2* and *RAD51C* etc. (3–6) HRR gene mutation results in DNA repair failures and increases lifelong breast cancer risks by 60–70% (7). Germline and somatic *BRCA1/2* mutations occur in 10–20% and 3–5% of all TNBC patients, respectively (1, 8), but the mutation pattern of the other HRR genes and the distribution of mutation sites is still uncovered.

Despite its role in oncogenesis, HRR gene mutation still has its silver lining. HRR gene mutation causes genomic loss of heterozygosity, telomeric allelic imbalance, and large-scale state transitions, named as homologous recombination deficiency (HRD) (9). Previous studies have demonstrated that *BRCA* mutations and HRD-positive status are associated with improved response rates to chemotherapy, but not necessarily with prolonged survival (10, 11). Moreover, *BRCA* mutation and HRD-positive status also acted as a potential predictor in platinum-containing chemotherapy efficacy (12–17). Yet, most of the findings on treatment response were based on neoadjuvant and metastatic settings, little was understood in the role of HRR gene mutations in early TNBC.

Furthermore, HRR gene mutation may have a potential role in guiding immunotherapy. Previous studies demonstrated that *BRCA1*-*PALB2* interaction disruption was positively associated with programmed cell death-ligand 1 (PD-L1) expression and T-lymphocyte infiltration in hepatocellular carcinoma (HCC) (18), and in ovarian cancer, DNA damage response-deficient (DDRD) breast tumors were associated with attenuated T cell inflammation (19, 20). However, the correlation of HRR gene mutation with immune infiltration, and their impact on the prognosis in TNBC patients have not been well elucidated. With the development of immune checkpoint inhibitors (ICIs) in TNBC (21, 22), understanding the association between HRR gene mutation, immune infiltration and PD-L1 expression, as well as their effect on prognosis, is crucial for guiding treatment and predicting prognosis in early TNBC patients.

Therefore, in the present study, we aimed to identify the mutation spectrum of HRR genes, and explore the association between HRR gene mutation and immune infiltration, as well as its impact on patient prognosis in early TNBC patients.

## Material and methods

### Patients and samples

For Ruijin Cohort 1, we retrospectively screened consecutive breast cancer patients treated at the Comprehensive Breast Health Center, Ruijin Hospital, Shanghai Jiao Tong University School of Medicine (RJBC-CBHC) from January 2012 to March 2019. Patients who met the following eligibility criteria were included: 1) invasive breast cancer, 2) pathologically diagnosed with TNBC, 3) available formalin fixed paraffin-embedded (FFPE) tissues. Exclusion criteria were as follows: 1) male breast cancer, 2) *de novo* stage IV, 3) incomplete immunohistochemistry (IHC) information.

For Ruijin Cohort 2, TNBC patients newly diagnosed and treated in RJBC-CBHC from June 2020 to January 2023 were prospectively recruited. Patients were eligible if they successfully received HRR genotyping. All tumor size, lymph node status, comorbidities, and adjuvant therapy strategies were permitted. Patients with metastatic or recurrent disease were excluded.

Archival FFPE blocks were selected from the biobank at the Department of Pathology, Shanghai Ruijin Hospital. Elaborate clinical data were retrieved from Shanghai Jiao Tong University Breast Cancer Database (SJTU-BCDB). All patients were given informed consent and our study was approved by the Ethical Committees of Ruijin Hospital, Shanghai Jiao Tong University School of Medicine (Clinical Trial Ethics Approval Number: 2016; 5–3). All procedures were in accordance with the 1964 Helsinki declaration and its later amendments.

### Ascertainment of clinicopathological information

At least two experienced pathologists (A. Li and M. Ruan) from the Department of Pathology, Ruijin Hospital, Shanghai Jiao Tong University School of Medicine, contributed to the tumor histopathological analysis. The IHC testing was used to determine the status of estrogen receptor (ER), progesterone receptor (PR), human epidermal growth factor receptor-2 (HER-2) and proliferation index (Ki-67). ER and PR positivity were defined as no less than 1% stained nuclei, as was described in our previous publications (23, 24). Ki67 ≥ 30% was classified as high-expression. HER-2 status was classified as “HER-2 Low” if IHC HER-2 1+ or HER-2 2+/Fluorescence *in situ* hybridization (FISH) negative, and HER-2 0 patients were defined as “HER-2 negative”.

### Follow-up

Patients from Ruijin Cohort 1 went through regular outpatient follow-ups or follow-up calls, once every three months within the first two year after surgery, once every six months through the third to the fifth year, and once every year ever since, in light of the American Society of Clinical Oncology Guidelines (ASCO guidelines) (25). Overall survival (OS) was defined as the time interval between surgery and death. Recurrence-free survival (RFS) was defined as the time interval between surgery and the event of local recurrence, distant metastasis or death. For patients with no events, OS and RFS were define as the time interval between surgery and the last follow-up date (Feb 12^th^ 2023).

### Targeted sequencing, bioinformatics analysis, and classification of variants

Genomic DNA from FFPE blocks was extracted, purified, and quantified using the MagPure FFPE DNA LQ Kit (Magen). A commercially available targeted AmoyDx HANDLE HRR NGS Panel covering 32 genes (17 HRR genes: *ATM*, *ATR*, *BARD1*, *BRCA1*, *BRCA2*, *BRIP1*, *CHEK1*, *CHEK2*, *FANCA*, *FANCL*, *MRE11*, *NBN*, *PALB2*, *RAD51B*, *RAD51C*, *RAD51D*, *RAD54L*; other cancer predisposition genes: *AR*, *BRAF*, *CDH1*, *CDK12*, *ERBB2*, *ESR1*, *HDAC2*, *HOXB13*, *KRAS*, *NRAS*, *PIK3CA*, *PPP2R2A*, *PTEN*, *STK11*, *TP53*) was used for next-generating sequencing analysis (Amoy Diagnostics, Xiamen, China). Prepared libraries were sequenced on the Illumina MiSeq system (Paired-End Reads, 2×150 cycles) and analyzed on the AmoyDx ANDAS Data Analyzer (Amoy Diagnostics, Xiamen, China) to accurately detect single nucleotide variants (SNVs) and short insertions/deletions (InDels) with detection sensitivity at variant allele frequency (VAF) ≥ 5%. The average coverage depth was 543X, and the results were further manually filtered to ensure that no false positives were reported.

### Immunohistochemistry and evaluation of immunostaining

The avidin-biotin-peroxidase complex method was adopted to perform immunohistochemistry (IHC) staining, as previously described (26, 27). After deparaffinization, rehydration and antigen retrieval, FFPE slides were incubated with CD8 (BD Pharmingen, US) and PD-L1 (Abcam, UK) primary antibodies overnight at 4°C. The secondary antibodies (Abcam, UK) were then applied and incubated at 37°C for an hour. After that, the slides were rinsed with phosphate buffer saline (PBS), stained with 3,3′-diaminobenzidine (DAB), and counterstained with hematoxylin. All specimens were independently and blindly evaluated by two expert pathologists (A. Li and M. Ruan). For CD8 staining, the number of positive cells was calculated in a 0.5 mm diameter cylinder and expressed as the mean value of the five fields per sample (cells per spot) (26). Individual values were used for correlation and survival analyses. Immunotype, as previously described (28), including “inflamed”, “immune excluded” and “immune desert”, was defined based on CD8^+^ T cell infiltration pattern at the tumor core and stroma. “Inflamed” represents diffuse CD8^+^ T cell infiltration in the tumor. “Immune excluded” is characterized by CD8^+^ T cell infiltration limited only to the invasive margin of the tumor. “Immune desert” stands for the absence of CD8^+^ T cell infiltration in the tumor. Tumor tissues with PD-L1^+^ tumor cells over 1% are considered PD-L1 positive. For combined survival analysis of HRR mutation and immune infiltration, patients were categorized into three groups: Group I, patients without HRR mutation and a low level of T cells; Group III, patients with HRR mutation and with a high level of T cells; Group II, the remaining patients (the average number of CD8^+^ T cells as cut-off value).

### Comparison with public reference controls

Data from the Cancer Genome Atlas (TCGA), the Molecular Taxonomy of Breast Cancer International Consortium (METABRIC), and the Memorial Sloan Kettering Cancer Center database 2018 (MSKCC 2018) was utilized to compare mutational frequencies. TCGA and MSKCC data was downloaded from the cBioPortal (29) (http://cbioportal.org/, files “TCGA, Firehose Legacy”, “MSK, Cancer cell 2018”). A total of 105 TNBC patients from the TCGA database and 225 TNBC MSK data were analyzed. METABRIC was retrieved as described previously (30), and 320 TNBC patients were used for analysis.

### Statistical analysis

The data distribution was characterized by frequency tabulation and summary statistics. Differences in categorical data was assessed through the Chi-square test or Fisher’s exact test. Kaplan-Meier curves and the Log-rank test were used to compare unadjusted survival between study groups. Cox proportional hazards models were used to evaluate hazard ratios across subgroups and to adjust for patients’ clinicopathological and therapeutic parameters. Two-sided *P* < 0.05 was considered statistically significant. All statistical analyses were performed using Stata version 16.1 (StataCorp LP, College Station, Texas).

## Results

### Patient and tumor characteristics

**Figure 1:**
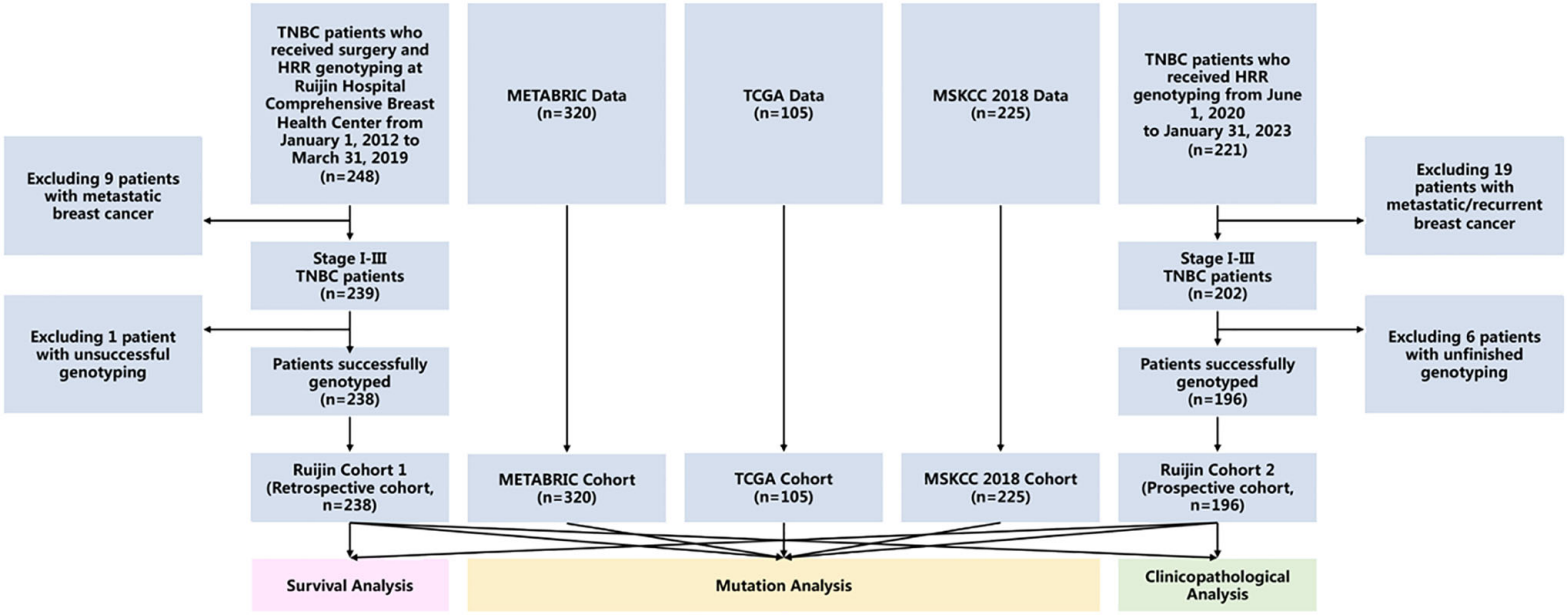
Study flowchart. Abbreviations: METABRIC, the Molecular Taxonomy of Breast Cancer International Consortium; MSKCC, the Memorial Sloan Kettering Cancer Center; TCGA, the Cancer Genome Atlas; TNBC, triple negative breast cancer.

A total of 238 and 196 TNBC patients were included in the Ruijin Cohort 1 and Ruijin Cohort 2, respectively. Public reference cohorts retrieved from cancer genome databases consist of 320 TNBC patients from METABRIC, 105 from TCGA, and 225 from MSKCC. All cohorts were used for mutation rate analysis. The pooled cohort (n = 434) of Ruijin Cohort 1 and Ruijin Cohort 2 was used for clinicopathological characteristics analysis, from which a subset of 383 patients with follow-up data was used for survival and predictive value of carboplatin treatment efficacy analysis (**Figure 1**).

Patient characteristics according to mutation status are shown in **Table 1**. Patients below 50 years accounted for 46.31% of the total cohort. Breast or ovarian cancer history was found in 63 (14.52%) patients, whereas 31 (7.14%) patients had a family history of other cancers. Twenty-three (5.30%) patients had at least one more primary malignancy other than breast cancer. Invasive ductal carcinoma (IDC) was observed in 84.56% of the study cohort, whereas the proportion of patients with a histological grade of III and a Ki-67 level above 30% were 67.74% and 76.04%, respectively. And HER-2 low status was seen in 55.07% of patients (**Table 1**).

**Table 1:** Patient and tumor characteristics based on mutation status.

	All Cases	HRR Mutation	Other Mutation†	Nonmutation Carriers	*P*
**Total Population**	434	95	256	83	
**Age**					0.167
≤50yr	201 (46.31)	50 (52.63)	109 (42.58)	42 (50.60)	
>50yr	233 (53.69)	45 (47.37)	147 (57.42)	41 (49.40)	
**Menopausal status**					0.059
Premenopausal	202 (46.54)	51 (53.68)	107 (41.80)	44 (53.01)	
Postmenopausal	232 (53.46)	44 (46.32)	149 (58.20)	39 (46.99)	
**Family history** **(Breast/ovarian cancer)**					0.082
No	371 (85.48)	75 (78.95)	221 (86.33)	75 (90.36)	
Yes	63 (14.52)	20 (21.05)	35 (13.67)	8 (9.64)	
**Family history (Other cancers)**					0.847
No	403 (92.86)	87 (91.58)	239 (93.36)	77 (92.77)	
Yes	31 (7.14)	8 (8.42)	17 (6.64)	6 (7.23)	
**Tumor Location**					0.925
Left	222 (51.15)	49 (51.58)	128 (50.00)	45 (54.22)	
Right	199 (45.85)	44 (46.32)	120 (46.88)	35 (42.17)	
Bilateral	13 (3.00)	2 (2.10)	8 (3.12)	3 (3.61)	
**Tumor pathology**					0.451
IDC	367 (84.56)	84 (88.42)	215 (83.98)	68 (81.93)	
Others*	67 (15.44)	11 (11.58)	41 (16.02)	15 (18.07)	
**Tumor Grade**					0.550
I-II	98 (22.58)	16 (16.84)	61 (23.83)	21 (25.30)	
III	294 (67.74)	71 (74.74)	170 (66.41)	53 (63.86)	
Unknown	42 (9.68)	8 (8.42)	25 (9.77)	9 (10.84)	
**cT stage**					0.133
T1	134 (30.88)	33 (34.74)	70 (27.34)	31 (37.35)	
T2	278 (64.06)	56 (58.94)	176 (68.75)	46 (55.42)	
T3	22 (5.07)	6 (6.32)	10 (3.91)	6 (7.23)	
**cN stage**					0.705
N0	198 (45.62)	46 (48.42)	117 (45.70)	35 (42.17)	
N+	236 (54.38)	49 (51.58)	139 (54.30)	48 (57.83)	
**HER-2**					0.463
Negative	195 (44.93)	48 (50.53)	111 (43.36)	36 (43.37)	
Low	239 (55.07)	47 (49.47)	145 (56.64)	47 (56.63)	
**Ki67**					0.141
≤30%	104 (23.96)	16 (16.84)	64 (25.00)	24 (28.92)	
>30%	330 (76.04)	79 (83.16)	192 (75.00)	59 (71.08)	
**Lymphovascular invasion**					0.769
No	369 (85.02)	83 (87.37)	216 (84.38)	70 (84.34)	
Yes	65 (14.98)	12 (12.63)	40 (15.62)	13 (15.66)	
**Second primary cancer**					0.796
No	411 (94.70)	90 (94.74)	241 (94.14)	80 (96.39)	
Yes	23 (5.30)	5 (5.26)	15 (5.86)	3 (3.61)	

†Other mutations: AR, BRAF, CDH1, CDK12, HDAC2, HOXB13, ERBB2, ESR1, KRAS, NRAS, PIK3CA, PPP2R2A, PTEN, STK11, TP53 mutations without HRR mutation.

*Other tumor pathology: invasive lobular carcinoma and special types of breast carcinoma.

HER-2, human epidermal growth factor receptor 2; HRR, homologous recombination repair; IDC, invasive ductal carcinoma.

### Frequency, distribution, and hotspots of tumor mutations

**Figure 2:**
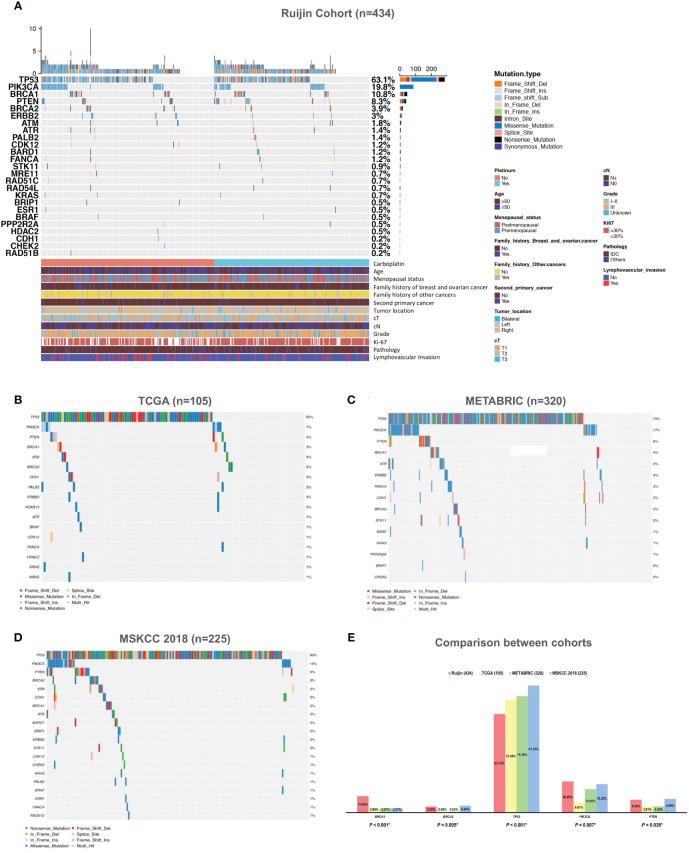
Mutation spectrum of HRR genes and other panel genes in TNBC. **(A)** Mutation spectrum of 434 TNBC patients from Ruijin Cohort. **(B)** Mutation spectrum of 105 TNBC patients from TCGA cohort. **(C)** Mutation spectrum of 320 TNBC patients from METABRIC cohort. **(D)** Mutation spectrum of 225 TNBC patients from MSKCC 2018 cohort. **(E)** Comparison between the prevalence of pathogenic variants of 17 homologous recombination repair genes across four cohorts. Abbreviations: HER-2, human epidermal growth factor receptor 2; HRR, homologous recombination repair; IDC, invasive ductal carcinoma; METABRIC, the Molecular Taxonomy of Breast Cancer International Consortium; MSKCC, the Memorial Sloan Kettering Cancer Center; PV, pathogenic variant; TCGA, the Cancer Genome Atlas; TNBC, triple negative breast cancer.

Tumor mutations in 17 HRR genes were detected in 21.89% (95/434) patients (**Figure 2A**). In Ruijin Cohort 1, *BRCA1* was the most frequently mutated HRR gene (10.08%), followed by *BRCA2* (5.88%), *ATR* (2.52%), *BARD1* (2.10%) and *PALB2* (2.10%) (**Table 2**). As for Ruijin Cohort 2, *BRCA1* was also the most frequently mutated HRR gene (11.22%), followed by *ATM* (2.04%) and *BRCA2* (1.53%) (**Table 2**). Notably, *BRCA1* was the most frequently mutated HRR gene across all cohorts (except MSKCC 2018), with a mutation rate ranging from 2.67% to 11.22% (**Figures 2A–D**). In MSKCC 2018, the most frequently mutated HRR gene was *ATM* (5.33%). The pathological variation (PV) prevalence of *BRCA1* was similar across public controls (2.67% to 2.86%), but was much higher (10.60%) in the study cohort, whereas the PV prevalence of *BRCA2* in our study (3.92%) and MSKCC 2018 (4.44%) greatly outnumbered that of TCGA (0.95%) and METABRIC (0.63%) (**Figure 2E**).

**Table 2:** Frequency of tumor mutations in TNBC patients.

Gene	Ruijin Cohort 1 (n=238)	Ruijin Cohort 2 (n=196)	TCGA Data (n=105)	METABRIC Data (n=320)	MSKCC 2018 Data (n=225)	*P*
Known HRR genes						
*BRCA1*	24 (10.08)	22 (11.22)	3 (2.86)	9 (2.81)	6 (2.67)	<0.001*
*BRCA2*	14 (5.88)	3 (1.53)	1 (0.95)	2 (0.63)	10 (4.44)	0.001*
*ATR*	6 (2.52)	0 (0.00)	0 (0.00)	2 (0.63)	5 (2.22)	0.037*
*BARD1*	5 (2.10)	0 (0.00)	0 (0.00)	0 (0.00)	3 (1.33)	0.012*
*PALB2*	5 (2.10)	1 (0.51)	0 (0.00)	0 (0.00)	3 (1.33)	0.040*
*ATM*	4 (1.68)	4 (2.04)	2 (1.90)	0 (0.00)	12 (5.33)	<0.001*
*FANCA*	3 (1.26)	2 (1.02)	0 (0.00)	1 (0.31)	2 (0.89)	0.652
*MRE11*	3 (1.26)	0 (0.00)	0 (0.00)	0 (0.00)	0 (0.00)	0.061
*RAD51C*	3 (1.26)	1 (0.51)	0 (0.00)	0 (0.00)	0 (0.00)	0.102
*RAD54L*	3 (1.26)	0 (0.00)	0 (0.00)	0 (0.00)	1 (0.44)	0.143
*BRIP1*	2 (0.84)	0 (0.00)	0 (0.00)	0 (0.00)	5 (2.22)	0.012*
*CHEK2*	2 (0.84)	0 (0.00)	0 (0.00)	0 (0.00)	3 (1.33)	0.107
*NBN*	1 (0.42)	0 (0.00)	0 (0.00)	0 (0.00)	0 (0.00)	0.705
*RAD51B*	1 (0.42)	0 (0.00)	0 (0.00)	0 (0.00)	0 (0.00)	0.705
*CHEK1*	0 (0.00)	0 (0.00)	0 (0.00)	0 (0.00)	0 (0.00)	NA
*FANCL*	0 (0.00)	0 (0.00)	0 (0.00)	0 (0.00)	0 (0.00)	NA
*RAD51D*	0 (0.00)	1 (0.51)	0 (0.00)	0 (0.00)	1 (0.44)	0.383
Other panel genes						
*TP53*	147 (61.76)	127 (64.80)	76 (72.38)	238 (74.38)	183 (81.33)	<0.001*
*PIK3CA*	56 (23.53)	31 (15.82)	7 (6.67)	48 (15.00)	41 (18.22)	0.002*
*PTEN*	19 (7.98)	17 (8.67)	4 (3.81)	13 (4.06)	20 (8.89)	0.075
*ERBB2*	9 (3.78)	4 (2.04)	0 (0.00)	4 (1.25)	13 (5.78)	0.006*
*CDK12*	5 (2.10)	1 (0.51)	1 (0.95)	0 (0.00)	3 (1.33)	0.057
*ESR1*	4 (1.68)	0 (0.00)	0 (0.00)	0 (0.00)	1 (0.44)	0.036*
*BRAF*	2 (0.84)	0 (0.00)	0 (0.00)	0 (0.00)	2 (0.89)	0.251
*KRAS*	2 (0.84)	1 (0.51)	1 (0.95)	3 (0.94)	2 (0.89)	1.000
*PPP2R2A*	2 (0.84)	0 (0.00)	0 (0.00)	0 (0.00)	0 (0.00)	0.251
*STK11*	2 (0.84)	2 (1.02)	0 (0.00)	4 (1.25)	6 (2.67)	0.377
*AR*	1 (0.42)	0 (0.00)	0 (0.00)	0 (0.00)	1 (0.44)	0.641
*CDH1*	1 (0.42)	0 (0.00)	2 (1.90)	6 (1.88)	17 (7.56)	<0.001*
*HDAC2*	1 (0.42)	1 (0.51)	0 (0.00)	0 (0.00)	0 (0.00)	0.463
*HOXB13*	0 (0.00)	0 (0.00)	0 (0.00)	0 (0.00)	0 (0.00)	NA
*NRAS*	0 (0.00)	1 (0.51)	0 (0.00)	0 (0.00)	0 (0.00)	0.278

Mutations in other cancer predisposition genes were detected in 59.91% (260/434) of patients. Both in Ruijin Cohort 1 and 2, *TP53* was the most frequently mutated panel gene (Ruijin Cohort 1: 61.76%; Ruijin Cohort 2: 64.80%), followed by *PIK3CA* (Ruijin Cohort 1: 23.53%; Ruijin Cohort 2: 15.82%), and *PTEN* (Ruijin Cohort 1: 7.98%; Ruijin Cohort 2: 8.67%) (**Table 2**). *TP53*, *PIK3CA*, and *PTEN* were also the most highly mutated gene across all cohorts, ranging from 55.88% to 81.33%, 6.67% to 23.53% and 3.81% to 8.89% of mutation rate in TCGA, METABRIC and MSKCC 2018, respectively (**Figures 2A–E**).

**Figure 3:**
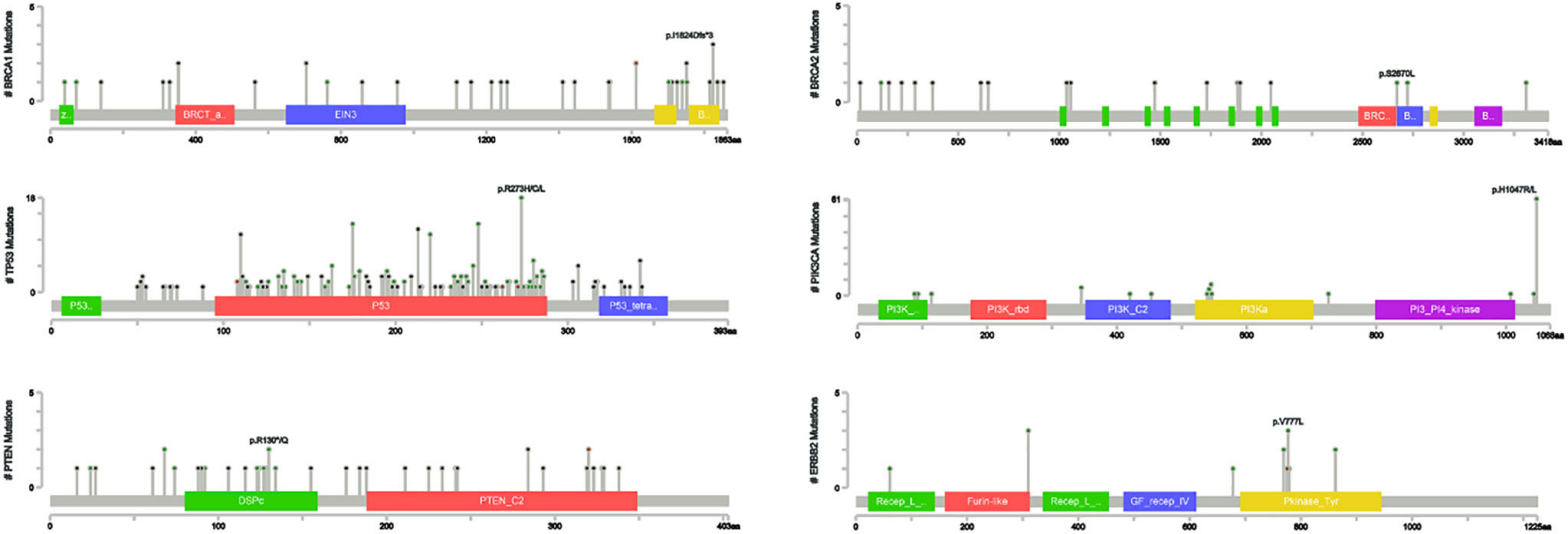
Distribution of mutation sites in *BRCA1*, *BRCA2*, *TP53*, *PIK3CA*, *PTEN* and *ERBB2*.

Locations of mutations and domains in proteins encoded by *BRCA1*, *BRCA2*, and other predisposition genes are shown by lollipop structures (**Figure 3**). The mutation hotspot of *BRCA1* lay at protein domain p.I1824Dfs*3, which accounts for 6.52% (3/46) of *BRCA1* mutations; and no mutation hotspot was detected for *BRCA2*. Mutations of *TP53* mainly located at *p.R273H/C/L* (6.57%, 18/274). The most frequently mutated protein domain of *PIK3CA* was *p.H1047R/L* (70.11%, 61/87). *PTEN* mutations most frequently occurred at p.R130*/Q (5.56%, 2/36). And *ERBB2* mutations mainly located on *p.V777L* (23.08%, 3/13). Further correlation analysis between HRR gene mutations indicated several co-mutation patterns, including *PALB2* and *RAD51B*, *NBN* and *RAD54L*, *ATR* and *RAD54B*, *ATR* and *RAD51L*, *FANCA* and *NBN*, *CHEK2* and *PPP2R2A*, and *PPP2R2A* and *RAD54L* (all odds ratios > 5.0, all *P* < 0.001) (**Supplementary Figure 1A** and **Figure 1B**).

### Association of HRR gene mutations with clinicopathological factors

In 434 patients from the pooled cohort of Ruijin Cohort 1 and Ruijin Cohort 2 with early TNBC, *BRCA* tumor mutation was associated with menopausal status (mutation rate 18.32% in premenopausal women versus 10.78% in postmenopausal women, *P* = 0.035), family history of breast/ovarian cancer (mutation rate 23.81% in women with family history versus 12.67% in women without family history, *P* = 0.016) and high Ki-67 levels (mutation rate 16.97% in high Ki-67 group versus 5.77% in low Ki-67 group, *P* = 0.005). *TP53* mutation was associated with high Ki-67 levels (mutation rate 68.18% in high Ki-67 group versus 47.12% in low Ki-67 group, *P* < 0.001). A significantly higher *PIK3CA* mutation rate was found in patients above 50 years (mutation rate 24.89% in patients above 50 years versus 14.43% in patients below 50 years, *P* = 0.007) and postmenopausal women (mutation rate 26.29% in postmenopausal women versus 12.87% in premenopausal women, *P* < 0.001), and was also associated with low tumor grade (mutation rate = 24.49% in grade I-II tumors versus 17.01% in grade III tumors, *P* = 0.049), HER-2 low status (mutation rate = 26.78% in HER-2 low tumors versus 11.79% in HER-2 negative tumors, *P* < 0.001) and low proliferation indices (mutation rate = 29.81% in low Ki-67 group versus 16.97% in high Ki-67 group, *P* = 0.004). No association between tumor mutation and secondary primary tumor was found in the study cohort (**Table 3**). Multivariate analysis further showed that breast/ovarian cancer family history (OR = 2.15, 95%CI 1.10–4.19, *P* = 0.025) and high Ki-67 levels (OR = 2.91, 95%CI 1.20–7.07, *P* = 0.018) were two independent predictive factors for *BRCA* mutation. A high Ki-67 level is also an independent predictive factor for *TP53* mutation (OR = 2.41, 95%CI 1.53–3.77, *P* < 0.001). Moreover, HER-2 low status was an independent predictive factor for *PIK3CA* mutation (OR = 2.37, 95%CI 1.37–4.08, *P* = 0.002) (**Supplementary Table 1**).

**Table 3:** Breast cancer characteristics among carriers of most frequently mutated genes.

	*BRCA* (n=61)	*P*	*TP53* (n=274)	*P*	*PIK3CA* (n=87)	*P*	*PTEN* (n=36)	*P*
**Age**		0.111		0.705		0.007*		0.020*
≤50yr	34 (55.74)		125 (45.62)		29 (33.33)		10 (27.78)	
>50yr	27 (44.26)		149 (54.38)		58 (66.67)		26 (72.22)	
**Menopausal status**		0.035*		0.760		<0.001*		0.097
Premenopausal	36 (59.02)		126 (45.99)		26 (29.89)		12 (33.33)	
Postmenopausal	25 (40.98)		148 (54.01)		61 (70.11)		24 (66.67)	
**Family history** **(Breast/ovarian cancer)**		0.016*		0.530		0.900		0.170
No	46 (75.41)		232 (84.67)		74 (85.06)		28 (77.78)	
Yes	15 (24.59)		42 (15.33)		13 (14.94)		8 (22.22)	
**Family history (Other cancers)**		0.418		0.320		0.715		0.734
No	55 (90.16)		257 (93.80)		80 (91.95)		33 (91.67)	
Yes	6 (9.84)		17 (6.20)		7 (8.05)		3 (8.33)	
**Tumor Location**		0.352		0.458		0.895		0.528
Left	36 (59.02)		134 (48.91)		44 (50.57)		18 (50.00)	
Right	23 (37.70)		131 (47.81)		41 (47.13)		16 (44.44)	
Bilateral	2 (3.28)		9 (3.28)		2 (2.30)		2 (5.56)	
**Tumor pathology**		0.356		0.527		0.065		0.097
IDC	54 (88.52)		234 (85.40)		68 (78.16)		27 (75.00)	
Others*	7 (11.48)		40 (14.60)		19 (21.83)		9 (25.00)	
**Tumor Grade**		0.372		0.176		0.049*		0.104
I-II	10 (16.39)		55 (20.07)		24 (27.59)		13 (36.11)	
III	46 (75.41)		196 (71.53)		50 (57.47)		19 (52.78)	
Unknown	5 (8.20)		23 (8.40)		13 (14.94)		4 (11.11)	
**cT stage**		0.421		0.299		0.060		0.365
T1	22 (36.07)		78 (28.47)		18 (20.69)		10 (27.78)	
T2	35 (57.38)		183 (66.79)		65 (74.71)		26 (72.22)	
T3	4 (6.56)		13 (4.74)		4 (4.60)		0 (0.00)	
**cN stage**		0.379		0.999		0.753		0.368
N0	31 (50.82)		125 (45.62)		41 (47.13)		19 (52.78)	
N+	30 (49.18)		149 (54.38)		46 (52.87)		17 (47.22)	
**HER-2**		0.121		0.239		<0.001*		0.070
Negative	33 (54.10)		129 (47.08)		23 (26.43)		11 (30.56)	
Low	28 (45.90)		145 (52.92)		64 (73.56)		25 (69.44)	
**Ki67**		0.005*		<0.001*		0.004*		0.028*
≤30%	6 (9.84)		49 (17.88)		31 (35.63)		14 (38.89)	
>30%	55 (90.16)		225 (82.12)		56 (64.37)		22 (61.11)	
**Lymphovascular invasion**		0.660		0.584		0.091		0.098
No	53 (86.89)		43 (15.69)		79 (90.80)		34 (94.44)	
Yes	8 (13.11)		231 (84.31)		8 (9.20)		2 (5.56)	
**Second primary cancer**		0.548		0.223		0.392		0.113
No	57 (93.44)		262 (95.62)		80 (91.95)		32 (88.89)	
Yes	4 (6.56)		12 (4.38)		7 (8.05)		4 (11.11)	

*Other tumor pathology: invasive lobular carcinoma and special types of breast carcinoma.

HER-2, human epidermal growth factor receptor 2; IDC, invasive ductal carcinoma.

### Prognostic values of HRR gene mutations

**Figure 4:**
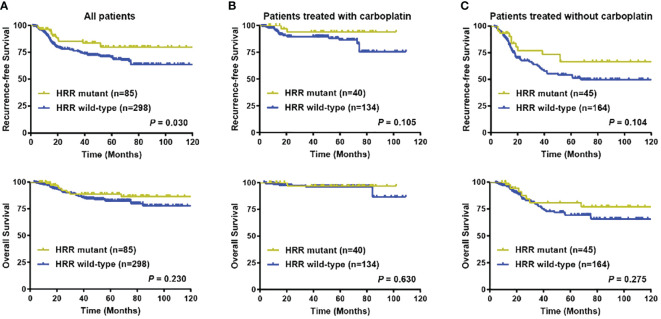
Prognostic significance of HRR gene mutation status. **(A)** Recurrence-free survival and overall survival in all patients according to HRR gene mutation status; **(B)** Recurrence-free survival and overall survival in patients treated with carboplatin chemotherapy according to HRR gene mutation status; **(C)** Recurrence-free survival and overall survival in patients treated without carboplatin chemotherapy according to HRR gene mutation status. Abbreviations: HRR, homologous recombination repair; OS, overall survival; RFS, recurrence-free survival.

After a median follow-up of 44.73 months, eighty-six (22.45%) out of 383 patients with follow-up data experienced with locoregional recurrence and (or) distant metastasis, whereas a total of 46 (12.01%) patients died. Univariate analysis indicated that 5-year RFS (79.88% versus 70.32%, *P* = 0.030), but not OS (88.99% versus 82.46%, *P* = 0.230) was better in HRR gene mutated patients compared with their wildtype counterparts (**Figure 4A**; **Supplementary Figure 2**). Yet after adjusting for age, tumor location, tumor grade, cT stage, cN stage, Ki67 level, lymphovascular invasion status, and carboplatin administration status, no difference in RFS (Adjusted *P* = 0.070) and OS (Adjusted *P* = 0.318) was observed between HRR gene mutated and non-mutated patients (**Supplementary Figures 3A, B**).

A total of 174 (45.43%) patients received carboplatin-containing adjuvant chemotherapy. Within the carboplatin treatment group, RFS events were found in 19 (10.92%) patients, while death occurred in 6 (3.45%) patients. In the rest 209 (54.57%) patients treated without carboplatin, locoregional recurrence and (or) distant metastasis was observed in 67 (32.06%) patients, whereas death was seen in 40 (19.14%) patients. Subgroup analysis further indicated that in patients treated with carboplatin chemotherapy, no survival difference was seen between the HRR genes mutation and non-mutation groups in 5-year RFS (93.84% versus 86.34%, *P* = 0.105) and OS (96.88% versus 96.39%, *P* = 0.630) (**Figure 4B**). And in subgroup without carboplatin treatment, no significant difference was found in terms of 5-year RFS (66.44% versus 52.74%, *P* = 0.104) and OS (80.97% versus 69.29%, *P* = 0.275) between HRR gene mutation carriers and non-carriers (**Figure 4C**).

### Survival benefit of carboplatin chemotherapy in HRR gene mutated and non-mutated groups

In the study cohort, a lower percentage of invasive ductal carcinoma (*P* = 0.026) and a higher frequency of unknown tumor grade (*P* = 0.003) was seen in the carboplatin treatment group than in the non-carboplatin treatment group, whereas Ki67 levels were higher in the carboplatin treatment group than in the non-carboplatin treatment group (*P* < 0.001). No difference was observed in other clinicopathological parameters (**Supplementary Table 2**). The 5-year RFS (88.17% versus 56.06%, *P* < 0.001) and OS (96.47% versus 72.15%, *P* < 0.001) in patients treated with carboplatin chemotherapy were significantly higher than those without (**Supplementary Figure 4A**). In HRR gene mutation carriers, superior 5-year RFS (93.84% versus 66.44%, *P* = 0.006) and OS (96.88% versus 80.97%, *P* = 0.030) were observed in the carboplatin treatment group compared with the non-carboplatin group (**Supplementary Figure 4B**). Patients without HRR gene mutations showed better 5-year RFS (86.34% versus 52.74%, *P* < 0.001) and OS (96.39% versus 69.29%, *P* < 0.001) when carboplatin was added to the treatment regimen (**Supplementary Figure 4C**). Furthermore, in multivariate analysis, carboplatin administration acted as an independent protective factor for RFS (HR = 0.23, 95%CI 0.13–0.38, *P* < 0.001) and OS (HR = 0.15, 95%CI 0.06–0.37, *P* < 0.001) in early TNBC (**Supplementary Figures 3A, B**).

**Figure 5:**
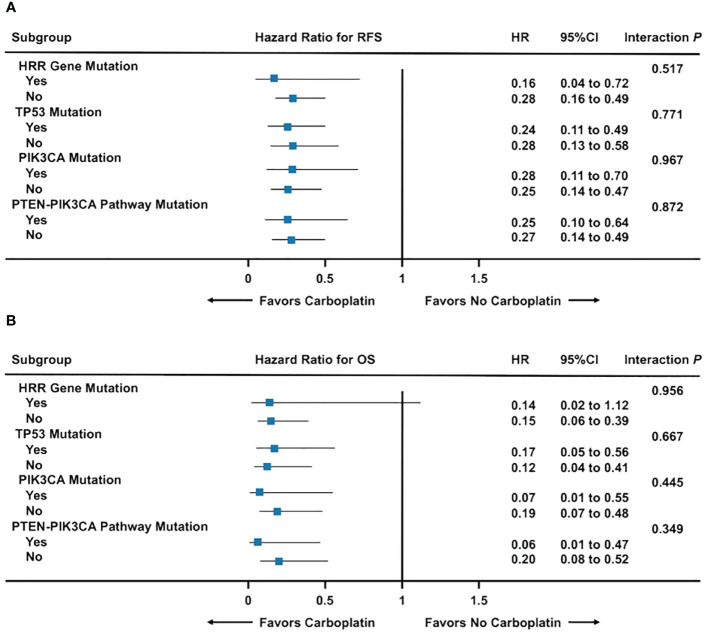
Forest plot of carboplatin chemotherapy efficacy among gene mutation subgroups. **(A)** Recurrence-free survival among gene mutation subgroups; **(B)** Overall survival among gene mutation subgroups. Abbreviations: HRR, homologous recombination repair; OS, overall survival; RFS, recurrence-free survival.

Across *TP53*, *PIK3CA*, and *PTEN-PIK3CA* pathway mutation and non-mutation subgroups, RFS and OS for early TNBC were significantly higher in patients treated with carboplatin chemotherapy than without regardless of gene mutation status (All Interaction *P* > 0.05) (**Figures 5A, B**; **Supplementary Figures 5C–F**). Nevertheless, survival benefit from carboplatin chemotherapy was only seen in *BRCA* mutation non-carriers. In *BRCA* mutation carriers, survival benefit from carboplatin chemotherapy was only observed in RFS (*P* = 0.046), but not OS (*P* = 0.077) (**Supplementary Figures 5A, B**).

### Association between HRR gene mutation, immune infiltration, and prognosis

**Figure 6:**
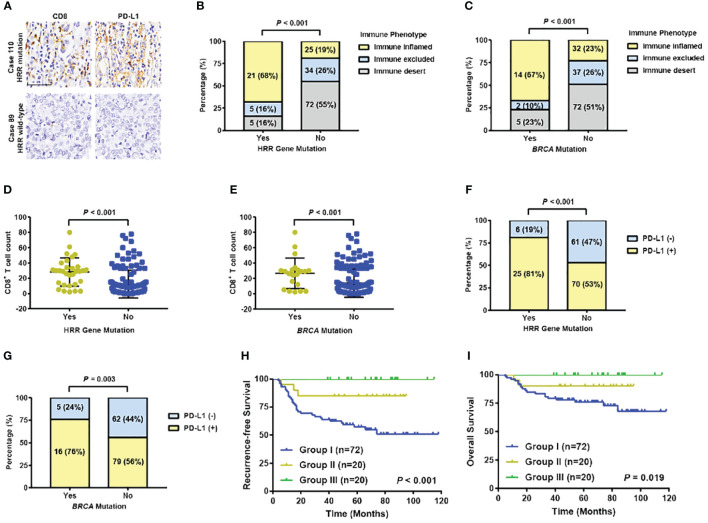
Association between HRR gene mutation and immune infiltration in TNBC patients. **(A)** Representative images of IHC staining of CD8 and PD-L1 in HRR-mutated and wildtype TNBC samples. (Up: high expression of CD8 and PD-L1; bottom: low expression of CD8 and PD-L1). Scale bar, 50 μm. **(B)** Distribution of immune phenotype by HRR gene mutation status. **(C)** Distribution of immune phenotype by *BRCA* mutation status. **(D)** Distribution of CD8^+^ T cell counts by HRR gene mutation status. **(E)** Distribution of CD8^+^ T cell counts by *BRCA* mutation status. **(F)** PD-L1 expression by HRR gene mutation status. **(G)** PD-L1 expression by *BRCA* mutation status. **(H)** Recurrence-free survival by HRR gene mutation status and CD8^+^ T cell counts (Group I: patients without HRR mutation and a low level of T cells; Group III: patients with HRR mutation and with a high level of T cells; Group II: the remaining patients). **(I)** Overall survival by HRR gene mutation status and CD8^+^ T cell counts (Group I: patients without HRR mutation and a low level of T cells; Group III: patients with HRR mutation and with a high level of T cells; Group II: the remaining patients). Abbreviations: HRR, homologous recombination repair; OS, overall survival; PD-L1, programmed cell death-ligand 1; RFS, recurrence-free survival; TNBC, triple-negative breast cancer.

In Ruijin Cohort 1, tumor specimens from 162 patients successfully achieved CD8 and PD-L1 immune staining and evaluation (**Figure 6A**). Forty-six patients were immune inflamed, 39 were immune exclude, and 77 were immune desert, respectively. Of 31 HRR gene mutated patients, 21 (68%) were immune inflamed, 5 (16%) were immune excluded, and 5 (16%) were immune desert, while in 131 HRR gene wildtype patients, 25 (19%) were immune inflamed, 34 (26%) were immune excluded, and 72 (55%) were immune desert (*P* < 0.001) (**Figure 6B**). Twenty-one patients were *BRCA* mutation carriers, consisting of 14 (67%) immune inflamed, 2 (10%) immune excluded, and 5 (23%) immune desert immunotypes, whereas within 141 noncarriers, the distribution of inflamed, immune excluded and immune desert immunotypes were 32 (23%), 37 (26%) and 72 (51%), respectively (*P* < 0.001) (**Figure 6C**). The level of immune infiltration and CD8^+^ T cell count in HRR gene or *BRCA* mutation carriers were significantly higher than their wildtype counterparts (both *P* < 0.001) (**Figures 6D, E**). Eighty one percent of HRR gene mutated tumors expressed PD-L1, which was 53% in HRR gene wildtype tumors (*P* < 0.001) (**Figure 6F**). In *BRCA* mutation carriers and non-carriers, 76% and 56% tumors expressed PD-L1 (*P* = 0.003), respectively (**Figure 6G**). Kaplan-Meier curves found that patients with both HRR gene mutation and high CD8^+^ T cell counts (average count as cut-off value) had the best RFS and OS, whereas patients without HRR gene mutation and low CD8^+^ T cell counts had the worst RFS (*P* < 0.001) and OS (*P* = 0.019) (**Figures 6H, I**).

## Discussion

In this large cohort of 434 Chinese patients with early TNBC, *BRCA1*, *BRCA2*, and *ATM* were the most frequently mutated HRR genes, and for other cancer predisposition genes, the top three ranking were *TP53*, *PIK3CA*, and *PTEN*, respectively. The mutation rate of *BRCA1*, *FANCA*, and *RAD51C* was higher in Ruijin Cohorts than in the TCGA, METABRIC and MSKCC 2018 cohorts. One reason lies in the difference of coverage depth. Deep coverage aids in differentiating sequencing errors from single nucleotide polymorphisms and increases accuracy in the final results. Based on previous studies, an average depth above 30X is required to detect all SNVs (31), and the depth of coverage in our study cohort, METABRIC (32), TCGA (33), and MSKCC 2018 (34) was 543X, 50X, 100X, and 771X, respectively, which was more than sufficient to achieve accurate results, especially in our study. Another reason was that data on public reference controls mainly report somatic mutations, while our study presented tumor mutations with next-generation target sequencing in FFPE samples, which may increase the mutation rate in some panel genes. The overall mutation rate of HRR genes was 21.89% in early TNBC, adding to a high clinical value and a great potential to carry out further research into HRR gene mutation’s association with clinicopathological parameters and survival prognosis.

Previous studies showed that the mutation hotspot of *BRCA1* was p.IGQ1824–1826*, which was p.I1824Dfs*3 in our study, indicating different types of mutations at the same location. And similar to our study, *BRCA2* also showed mutations on sporadic sites without a detected hotspot (35). The most frequently mutated domain of *TP53* was *p.R175H* according to Kim Y et al (36), *p.R248Q* according to Jouali F et al (37), but *p.R273H/C/L* in our study. The mutation hotspot of *PIK3CA* was *p.H1047R/L* in our cohort, which is in line with Martínez-Sáez O et al’s and Jouali F et al’s studies (38, 39). *PTEN* mutations most frequently occurred at *p.K60SfsX98* (40), which was p.R130*/Q in the Ruijin cohort. And *ERBB2* mutation hotspot was located at *p.V777L* in our study, which was *p.V842I* according to Robichaux JP et al’s study (41). Collectively, these detected mutation hotspots in our study still require further exploration to verify their biological functions. In terms of the association between gene mutation status and clinicopathological factors, *BRCA* mutation was associated with high proliferation indices, probably due to the fact that *BRCA* mutated cancers are more likely to show basal-like phenotypes (42). *PIK3CA* mutations, on the contrary, shows a tendency towards low proliferation indices, indicating that the mutation of *BRCA* and *PIK3CA* might be mutually exclusive, which was in line with previous studies (43, 44). The mechanism under this mutually exclusiveness is still uncovered, and further investigations are still needed.

In survival prognosis analysis, tumor mutations in HRR genes didn’t have significant impact on RFS and OS, which was similar with Punie K et al. ‘s study, indicating that there was no significant survival difference between germline *BRCA* mutated and non-mutated breast cancer. Similar results with the GeparSixto trial, all the patients received chemotherapy in our cohort, and *BRCA* mutation didn’t significantly affect survival prognosis in early TNBC patients, probably due to the DNA damage chemotherapeutic agents (13). Although the prognosis of patients with HRR genes mutation carriers and non-carriers in early TNBC were similar, future studies are needed to identify certain gene combinations within the HRR pathway where mutation status could predict survival.

Moreover, carboplatin-containing chemotherapy could significantly improve response rates in TNBC patients, according to GeparSixto trial (13), indicating carboplatin is an effect agent in TNBC. However, it remains uncertain whether carboplatin could improve prognosis in early TNBC patients and patients with HRR gene mutation. In our study, carboplatin treatment significantly improved survival prognosis regardless of HRR gene, *TP53* and *PIK3CA* mutation status and acted as an independent protective factor for RFS and OS. This result might derive from the fact that most of the patients received carboplatin chemotherapy sequenced with anthracyclines and cyclophosphamide, all of which were DNA damaging agents and could cast severe DNA damage to tumor cells when being used together (45). When the degree of damage exceeded the DNA repair capacity of the HRR pathway, cell death could occur regardless of HRR status, which might be the possible molecular mechanism in our study. Although no difference in carboplatin efficacy was observed between HRR gene mutation carriers and non-carriers, HRR gene mutation’s prognostic role in patients treated with other agents, such as PARP inhibitors (46), still requires further investigation.

Furthermore, based on our study, immune infiltration and PD-L1 expression was positively associated with HRR gene and *BRCA* mutation, which was in line with the results in liver and ovarian cancers (18, 19). According to previous researches, the underlying mechanism of this phenomenon could be listed as follows. On one hand, the stimulator of interferon genes (STING) pathway, which plays a crucial role in innate immune response to cancer, can be activated at DNA damage repair (DDR) deficiency and exposure to DNA-damaging agents (47), which stimulates interferon (IFN) production and in turn recruit T lymphocytes through the STING-interferon regulatory factor 3 (IRF3) pathway (48, 49), leading to T cell infiltration in HRR gene mutated tumors. On the other hand, the activated STING pathway induces PD-L1 expression through the STING-IRF3-signal transducer and activator of transcription 1 (STAT1) pathway, while DNA damage further upregulates *PD-L1* mRNA expression, leading to increased PD-L1 expression in HRR mutation carriers (50–52). Additionally, patients with both HRR gene mutation and high immune infiltration has the superior disease outcome in our study. Potential mechanism might lie in that tumors with HRR gene mutation have deficiency in repairing DSBs caused by chemotherapy, whereas damaged tumor cells become more recognizable and vulnerable to immunologic cytotoxicity caused by infiltrating T lymphocytes (20). Future researches are still needed to further identify the underlying mechanisms.

There are potential limitations to this study. The cohort in which we analyzed survival prognosis is a single-center retrospective cohort, including a continuous cohort of triple negative breast cancer patients who underwent surgery, which might be susceptible to selection bias and lack of representativeness and requires further validation in further multicenter prospective studies. In addition, tumor-specific somatic mutations can be further distinguished from tumor mutations in FFPE samples by stroking out background germline mutations, which can be achieved through targeted-NGS in peripheral blood or normal breast tissues. Besides, new generation method in assessing immune infiltration status, such as multiple immunofluorescences, spatial transcriptomics, might help to provide more precise classification. Furthermore, though HRR mutation status could not predict carboplatin efficacy, there might be a better model, to name a few, HRD score (12), Neo-Family History Score (53), and ten HRR gene-model (17) to differentiate survival prognosis between platinum-containing treatment groups and their counterparts, which needs further biomarker studies in the future.

## Conclusion

High frequency of tumor mutations in HRR genes was found in early TNBC patients, but showed no significant association with survival outcome, regardless of carboplatin treatment. Immune infiltration and PD-L1 expression was positively associated with HRR mutation, and patients with both HRR mutation and high CD8^+^ T cell infiltration levels had superior disease outcome. Further genetic testing implication and novel clinical trials with specific targeted HRR pathway therapies need to be investigated.

## Data availability statement

The original contributions presented in the study are included in the article/**Supplementary Material**. Further inquiries can be directed to the corresponding authors.

## Ethics statement

The studies involving humans were approved by Ethical Committees of Ruijin Hospital, Shanghai Jiao Tong University School of Medicine. The studies were conducted in accordance with the local legislation and institutional requirements. The human samples used in this study were acquired from a by- product of routine care or industry. Written informed consent for participation was not required from the participants or the participants’ legal guardians/next of kin in accordance with the national legislation and institutional requirements.

## Author contributions

ZW: Writing – review & editing, Writing – original draft, Project administration, Methodology, Investigation, Funding acquisition, Data curation. AL: Writing – review & editing, Writing – original draft, Validation, Resources, Methodology, Investigation, Formal analysis, Data curation. YL: Writing – review & editing, Writing – original draft, Visualization, Software, Methodology, Data curation, Conceptualization. MH: Writing – review & editing, Visualization, Software. MR: Writing – review & editing, Resources, Methodology, Formal analysis. CW: Writing – review & editing, Validation, Supervision, Resources, Methodology. XZ: Writing – review & editing, Resources, Methodology, Formal analysis. CZ: Writing – review & editing, Validation, Software, Resources, Methodology, Formal analysis. KS: Writing – review & editing, Validation, Supervision, Resources, Project administration, Funding acquisition, Data curation, Conceptualization. LD: Writing – review & editing, Supervision, Project administration, Conceptualization. XC: Writing – review & editing, Validation, Supervision, Resources, Project administration, Methodology, Funding acquisition, Data curation.
