# Supplementary material for: Association of tumor immune infiltration and prognosis with homologous recombination repair genes mutations in early triple-negative breast cancer
Source: Front Immunol. 2024 Jul 4;15:1407837. doi: 10.3389/fimmu.2024.1407837 (PMC11254810; doi:10.3389/fimmu.2024.1407837)
Supplement: Supplementary file 1 [file DataSheet_1.docx]

**Supplementary Table 1:**

**Multivariate analysis of clinicopathological parameters among carriers of most frequently mutated genes.**

| **Clinicopathological parameters** | **Odds ratio** | **95% Confidence Interval** | ***P*** |
| --- | --- | --- | --- |
| ***BRCA* mutation** |  |  |  |
| Postmenopausal versus Premenopausal | 0.61 | 0.35-1.08 | 0.088 |
| Family history versus No family history of Breast/Ovarian cancer | 2.15 | 1.10-4.19 | 0.025* |
| Ki67>30% versus Ki67≤30% | 2.91 | 1.20-7.07 | 0.018* |
| ***TP53* mutation** |  |  |  |
| Ki67>30% versus Ki67≤30% | 2.41 | 1.53-3.77 | <0.001* |
| ***PIK3CA* mutation** |  |  |  |
| Age>50 versus Age≤50 | 0.89 | 0.38-2.07 | 0.779 |
| Postmenopausal versus Premenopausal | 2.10 | 0.88-5.00 | 0.094 |
| Tumor Grade III versus I-II | 0.93 | 0.48-1.81 | 0.841 |
| HER-2 low versus HER-2 negative | 2.37 | 1.37-4.08 | 0.002* |
| Ki67>30% versus Ki67≤30% | 0.69 | 0.37-1.29 | 0.245 |
| ***PTEN* mutation** |  |  |  |
| Age>50 versus Age≤50 | 2.14 | 0.99-4.62 | 0.053 |
| Ki67>30% versus Ki67≤30% | 0.54 | 0.26-1.11 | 0.092 |

**Supplementary Table 2:**

**Patient and tumor characteristics based on carboplatin administration status in Ruijin Cohort.**

|  | **Carboplatin (n=205)** | **No carboplatin (n=229)** | ***P*** |
| --- | --- | --- | --- |
| **Age** |  |  | 0.329 |
| ≤50yr | 100 (48.78) | 101 (44.10) |  |
| >50yr | 105 (51.22) | 128 (55.90) |  |
| **Menopausal status** |  |  | 0.910 |
| Premenopausal | 96 (46.83) | 106 (46.29) |  |
| Postmenopausal | 109 (53.17) | 123 (53.71) |  |
| **Family history (Breast/ovarian cancer)** |  |  | 0.376 |
| No | 172 (83.90) | 199 (86.90) |  |
| Yes | 33 (16.10) | 30 (13.10) |  |
| **Family history (Other cancers)** |  |  | 0.810 |
| No | 191 (93.17) | 212 (92.58) |  |
| Yes | 14 (6.83) | 17 (7.42) |  |
| **Tumor Location** |  |  | 0.417 |
| Left | 108 (52.68) | 114 (49.78) |  |
| Right | 89 (43.41) | 110 (48.03) |  |
| Bilateral | 8 (3.90) | 5 (2.18) |  |
| **Tumor pathology** |  |  | 0.026* |
| IDC | 165 (80.49) | 202 (88.21) |  |
| Others* | 40 (19.51) | 27 (11.79) |  |
| **Tumor Grade** |  |  | 0.003* |
| I-II | 37 (18.05) | 61 (26.64) |  |
| III | 139 (67.80) | 155 (67.69) |  |
| Unknown | 29 (14.15) | 13 (5.68) |  |
| **cT stage** |  |  | 0.981 |
| T1 | 63 (30.73) | 71 (31.00) |  |
| T2 | 132 (64.39) | 146 (63.76) |  |
| T3 | 10 (4.88) | 12 (5.24) |  |
| **cN stage** |  |  | 0.496 |
| N0 | 90 (43.90) | 108 (47.16) |  |
| N+ | 115 (56.10) | 121 (52.84) |  |
| **HER-2** |  |  | 0.086 |
| Negative | 101 (49.27) | 94 (41.05) |  |
| Low | 104 (50.73) | 135 (58.95) |  |
| **Ki67** |  |  | <0.001* |
| ≤30% | 30 (14.63) | 74 (32.31) |  |
| >30% | 175 (85.37) | 155 (67.69) |  |
| **Lymphovascular invasion** |  |  | 0.071 |
| No | 181 (88.29) | 188 (82.10) |  |
| Yes | 24 (11.71) | 41 (17.90) |  |
| **Second primary cancer** |  |  | 0.424 |
| No | 196 (95.61) | 215 (93.89) |  |
| Yes | 9 (4.39) | 14 (6.11) |  |

*Other tumor pathology: invasive lobular carcinoma and special types of breast carcinoma

Abbreviations: HER-2, human epidermal growth factor receptor 2; IDC, invasive ductal carcinoma
