# Supplementary material for: Association of tumor immune infiltration and prognosis with homologous recombination repair genes mutations in early triple-negative breast cancer
Source: Front Immunol. 2024 Jul 4;15:1407837. doi: 10.3389/fimmu.2024.1407837 (PMC11254810; doi:10.3389/fimmu.2024.1407837)
Supplement: Supplementary file 2 [file DataSheet_2.docx]

**Supplementary Figure 1:**

**Correlation between gene mutations found in early TNBC patients.**

**
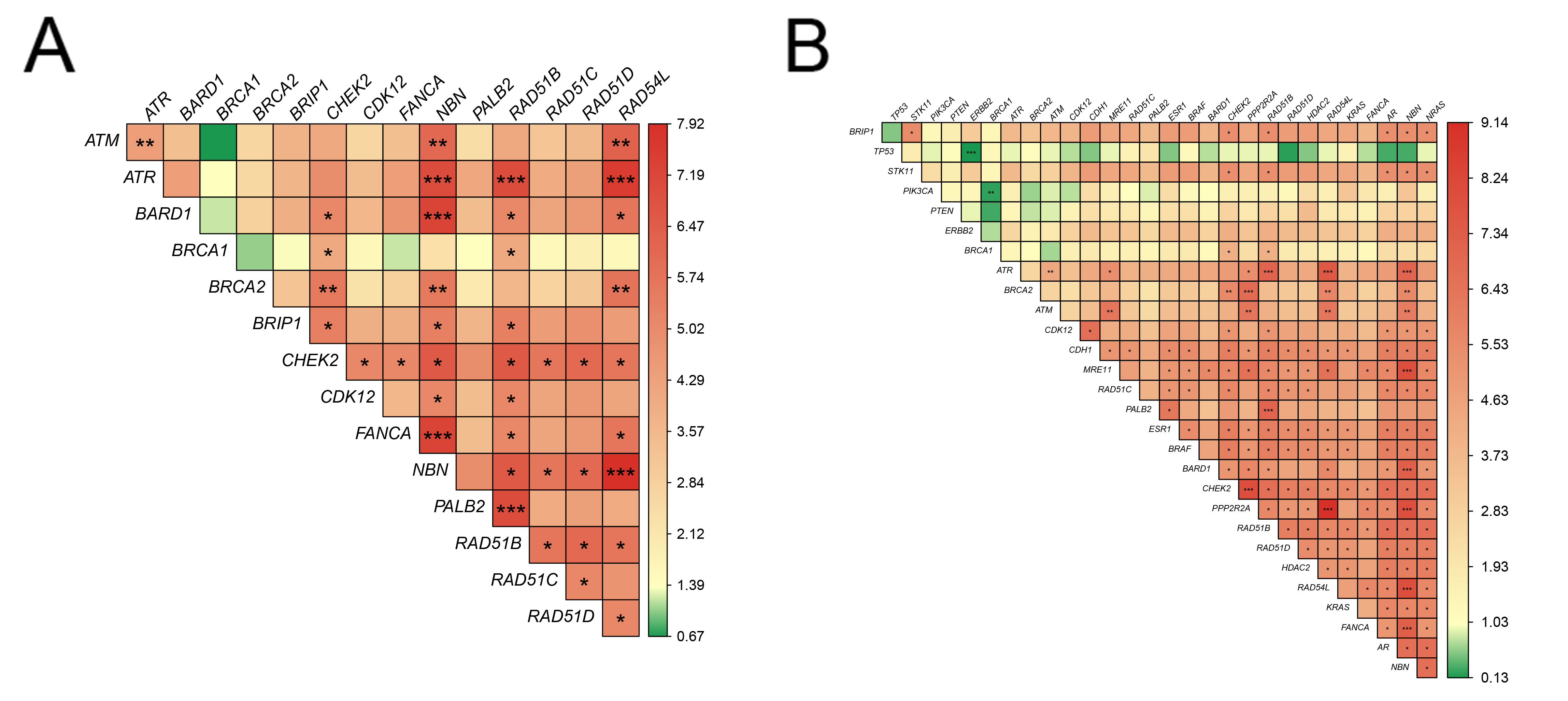
**

(A) Correlation between 15 HRR gene mutations. (B) Correlation between 29 HRR and other panel gene mutations. Correlation coefficients are indicated by the color gradient as indicated. *, *P* < 0.05; **, *P* < 0.01; ***, *P* < 0.001.

Abbreviations: HRR, homologous recombination repair; TNBC, triple-negative breast cancer.

**Supplementary Figure 2:**

**Prognostic significance of *BRCA*, *TP53* and *PIK3CA* mutation status.**

**
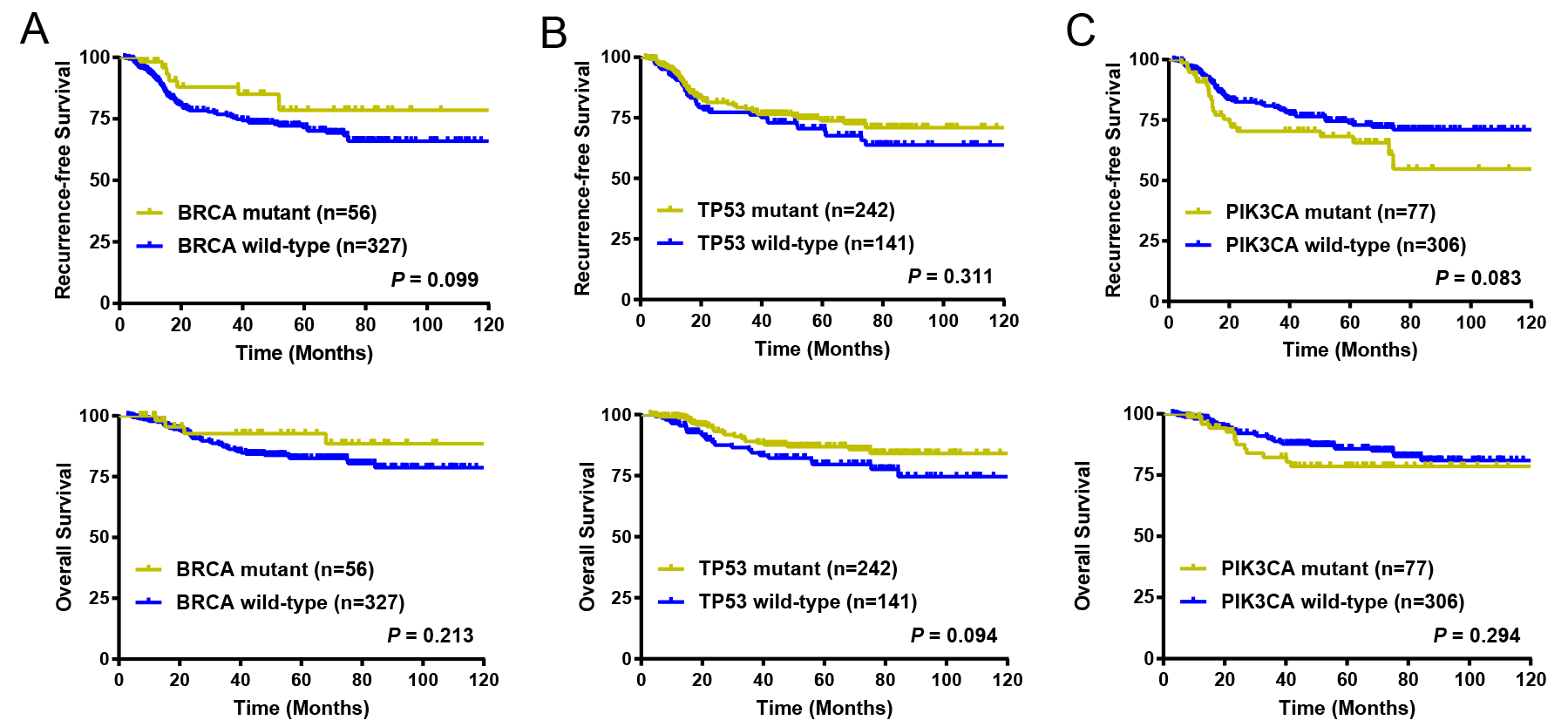
**

(A) Recurrence-free survival and overall survival according to *BRCA* mutation status; (B) Recurrence-free survival and overall survival according to *TP53* mutation status; (C) Recurrence-free survival and overall survival according to *PIK3CA* mutation status.

**Supplementary Figure 3:**

**
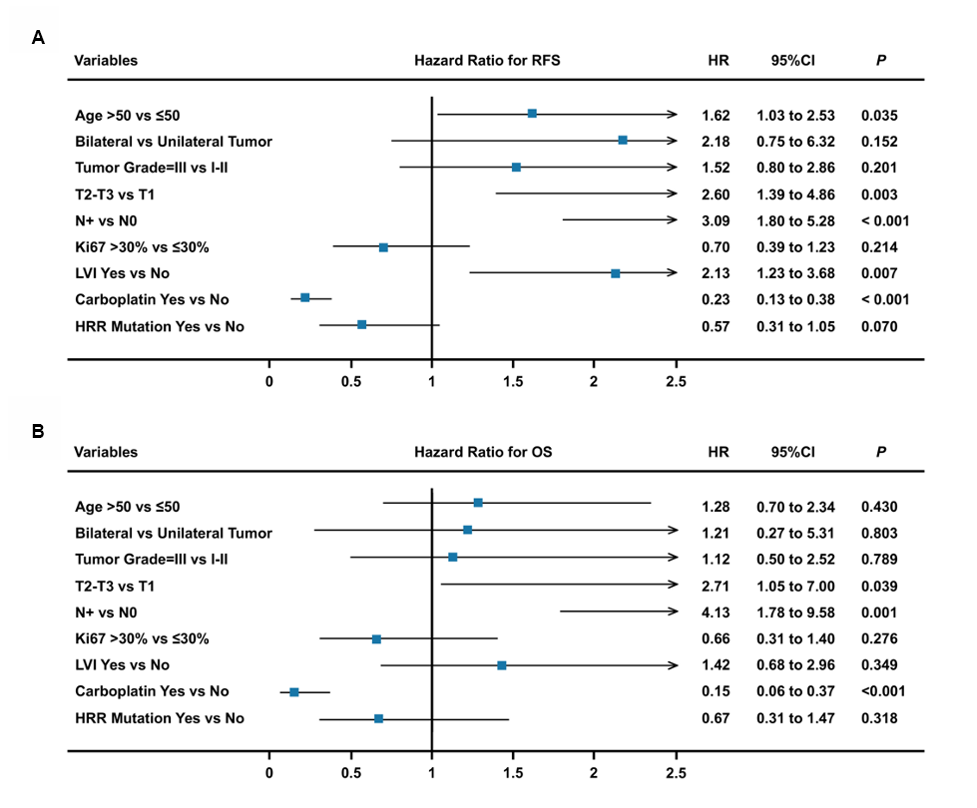
Multivariate analysis.**

(A) Multivariate analysis for recurrence-free survival. (B) Multivariate analysis for overall survival.

Abbreviations: HRR, homologous recombination repair; LVI, lymphovascular invasion; OS, overall survival; RFS, recurrence-free survival.

**Supplementary Figure 4:**

**Prognostic significance of carboplatin chemotherapy.**

**
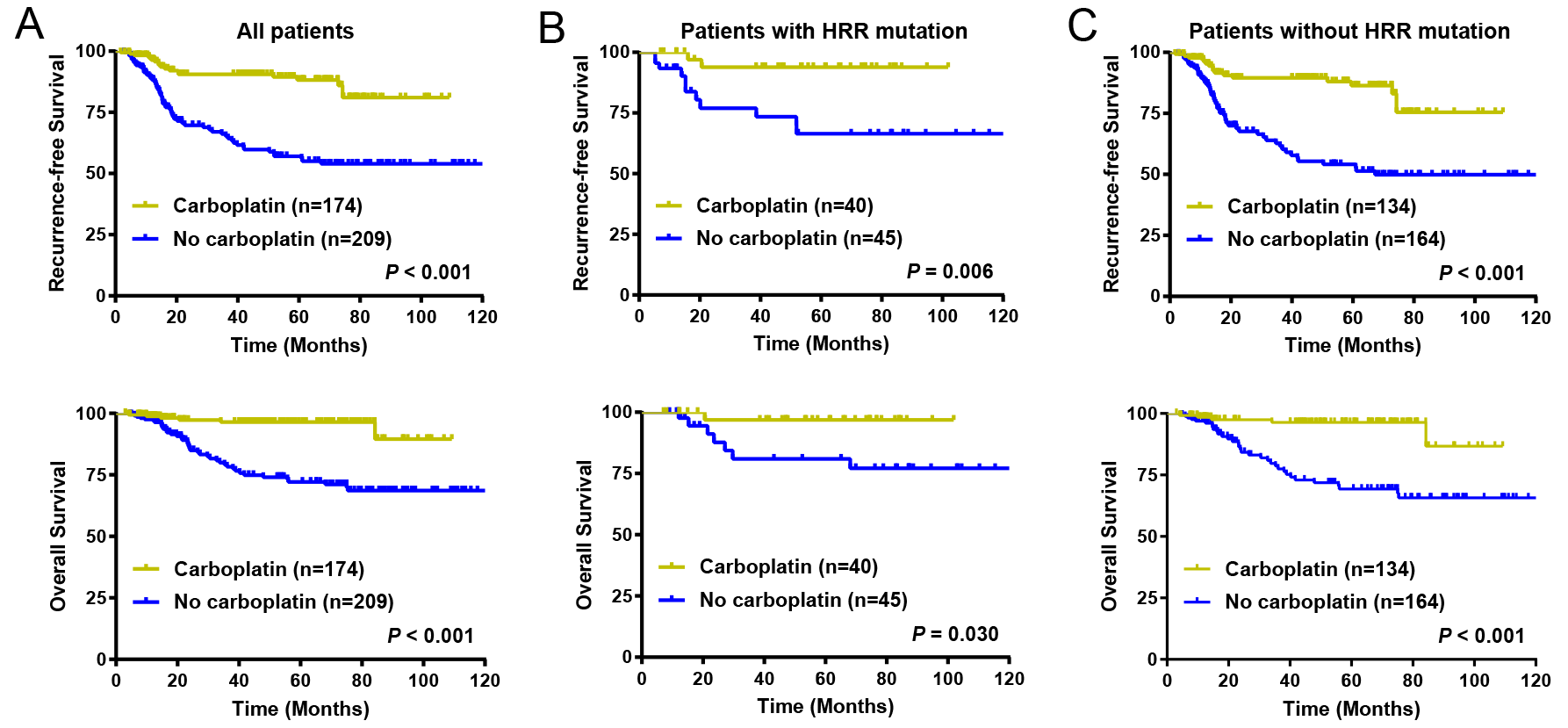
**

(A) Recurrence-free survival and overall survival in all patients according to carboplatin chemotherapy; (B) Recurrence-free survival and overall survival in patients with HRR gene mutation according to carboplatin chemotherapy; (C) Recurrence-free survival and overall survival in patients without HRR gene mutation according to carboplatin chemotherapy.

Abbreviations: HRR, homologous recombination repair; OS, overall survival; RFS, recurrence-free survival.

**Supplementary Figure 5:**

**Prognostic significance of carboplatin chemotherapy in patients with and without *BRCA, TP53 and PIK3CA* mutation.**

**
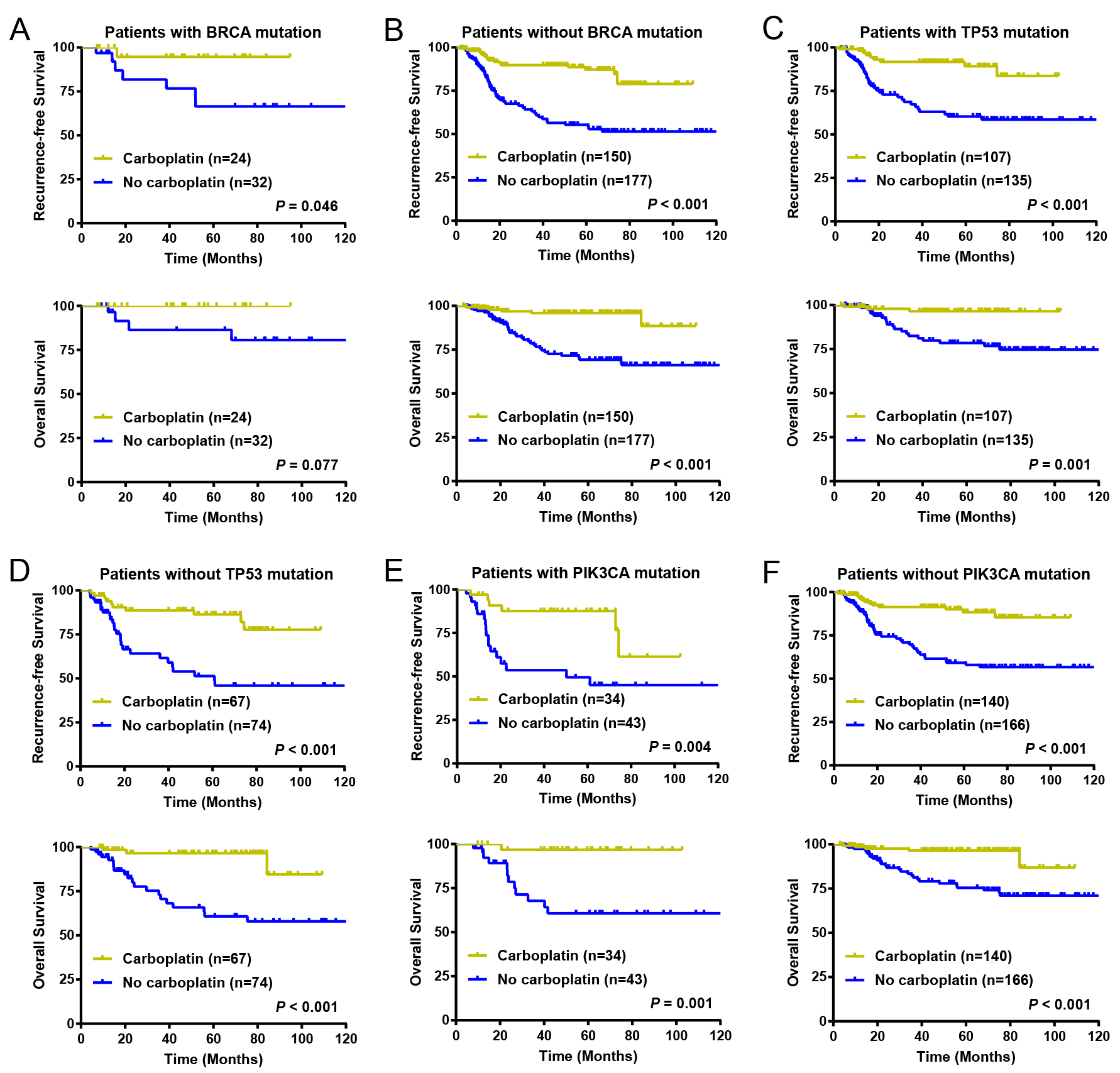
**

(A) Recurrence-free survival and overall survival in patients with *BRCA* mutation according to carboplatin chemotherapy; (B) Recurrence-free survival and overall survival in patients without *BRCA* mutation according to carboplatin chemotherapy; (C) Recurrence-free survival and overall survival in patients with *TP53* mutation according to carboplatin chemotherapy; (D) Recurrence-free survival and overall survival in patients without *TP53* mutation according to carboplatin chemotherapy; (E) Recurrence-free survival and overall survival in patients with *PIK3CA* mutation according to carboplatin chemotherapy; (F) Recurrence-free survival and overall survival in patients without *PIK3CA* mutation according to carboplatin chemotherapy.
